# Supplementary material for: A somatic role for the histone methyltransferase Setdb1 in endogenous retrovirus silencing
Source: Nat Commun. 2018 Apr 27;9:1683. doi: 10.1038/s41467-018-04132-9 (PMC5923290; doi:10.1038/s41467-018-04132-9)
Supplement: Supplementary file 1 — Supplementary Information [file 41467_2018_4132_MOESM1_ESM.pdf]

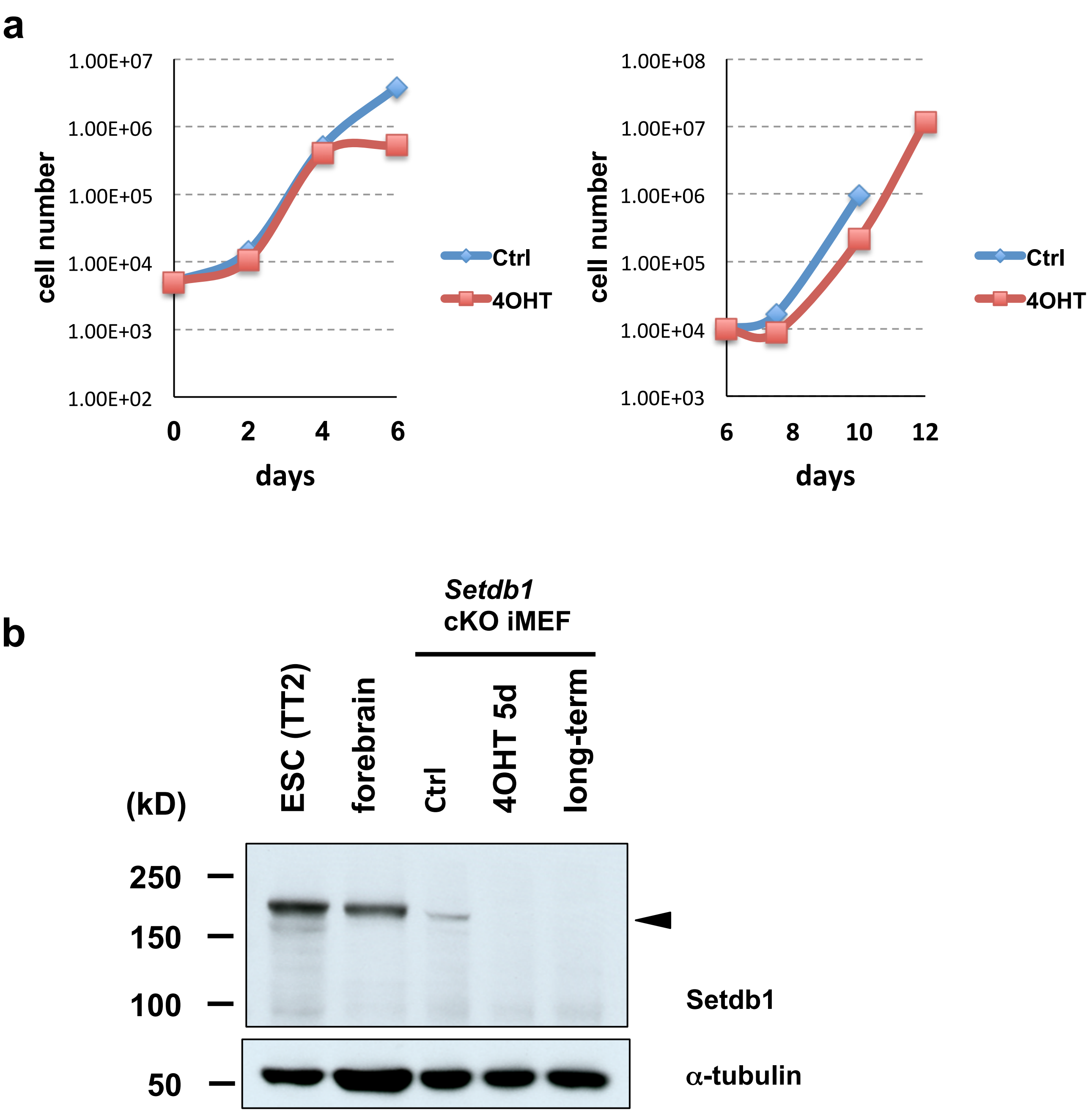

Supplementary Figure 1. iMEFs after depletion of Setdb1 shows temporal growth defect. (a) *Setdb1* cKO iMEFs (MEF3-12) were treated 4OHT for first 4 days. At day 6, cells were trypsinized and replated. (**b)** The amount of Setdb1 protein in ESCs, fetal forebrain (E14.5) and *Setdb1* cKO iMEFs (without (ctrl), after 4OHT treatment for 5 days (4OHT 5d) and cultured more than 2 weeks after 4OHT treatment (long-term)).

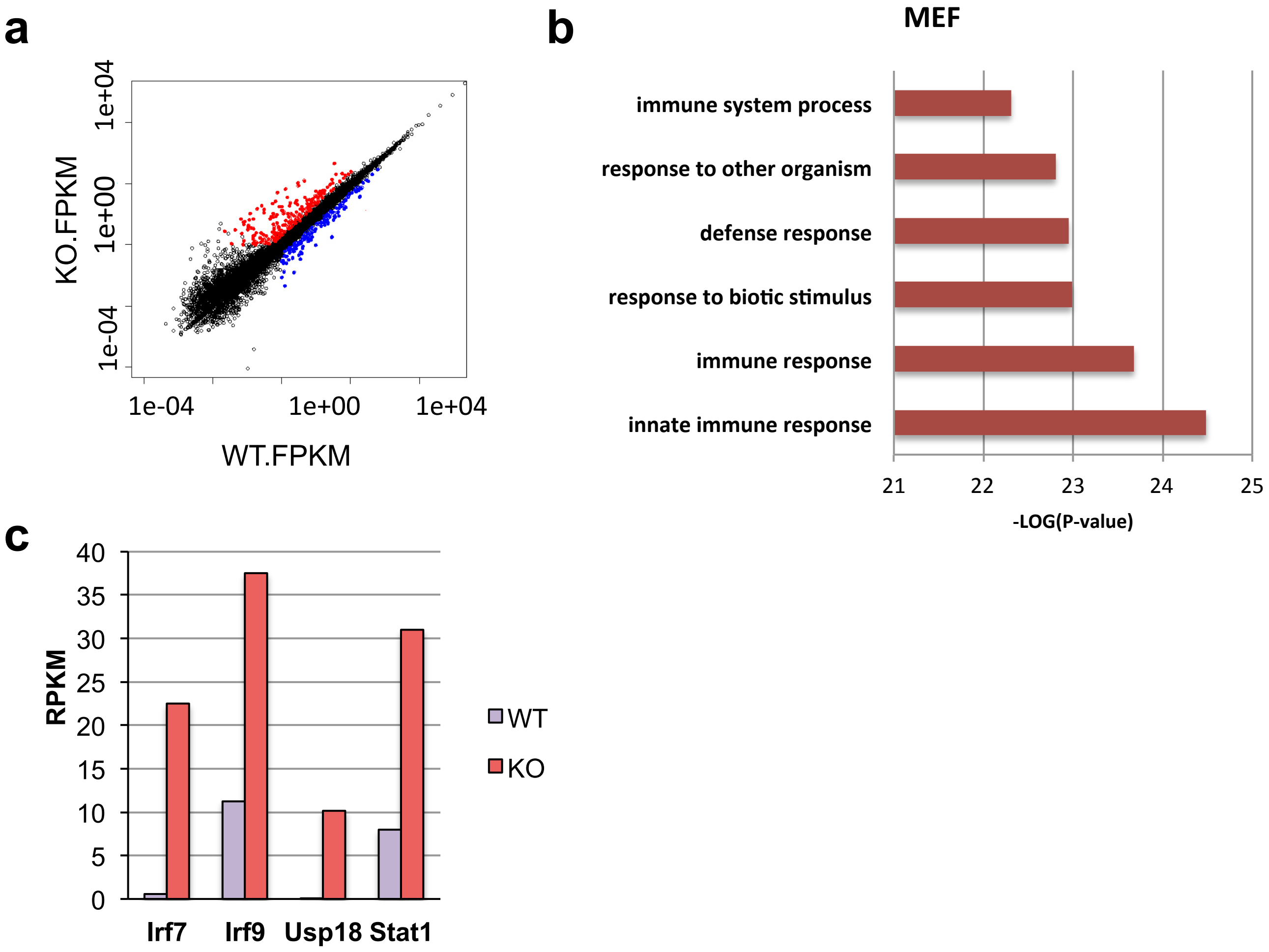

Supplementary Figure 2. Gene expression following *Setdb1* KO (**a**) Differential gene expression analysis of RefSeq genes (29,107 total) in *Setdb1* cKO iMEFs untreated or treated with 4OHT for 5 days. Upregulated (red) and downregulated genes (blue) showing fold change > 2. (**b**) GO term analysis of the upregulated genes of *Setdb1* cKO iMEF with 4OHT (**c**) Expression of representative genes of IFN pathway

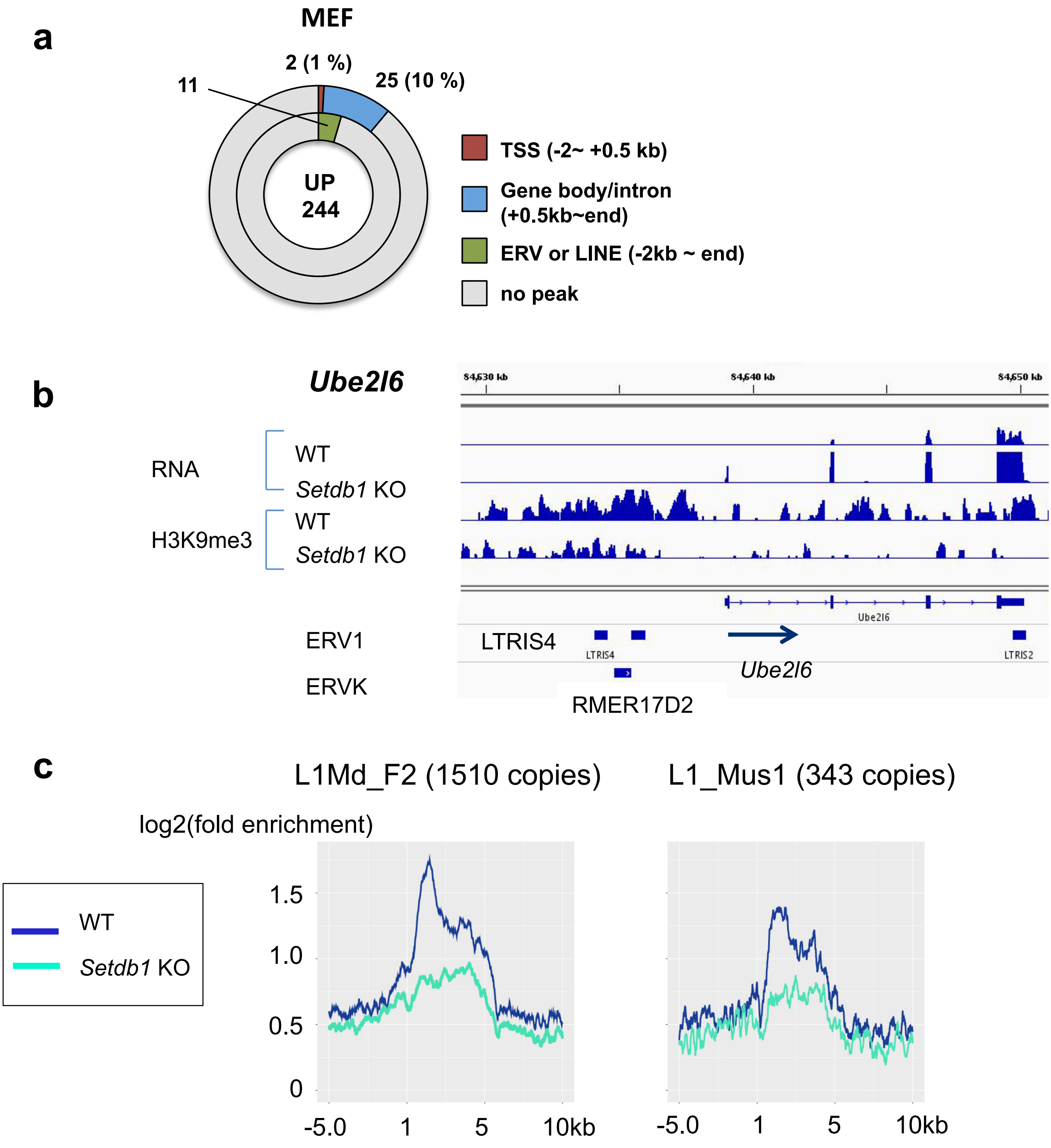

Supplementary Figure 3. Correlation between upregulated genes and H3K9me3 mark (**a**) The overlap of upregulated genes (244) and H3K9me3 marked genes (27) is shown, along with genes which ERVs and LINEs are located nearby (-2kb~end) in iMEF. (**b**) RNA-seq and ChIP-seq read density at *Ube2l6* gene in iMEFs. (**c**) ChIP-seq read densities for H3K9me3 around LINE families Full length elements of L1Md_F2 and L1_Mus1 families are aligned. Depletion of Setdb1 in iMEFs leads to distinct reduction in H3K9me3 in LINE regions. Read counts are normalized with million mapped reads.

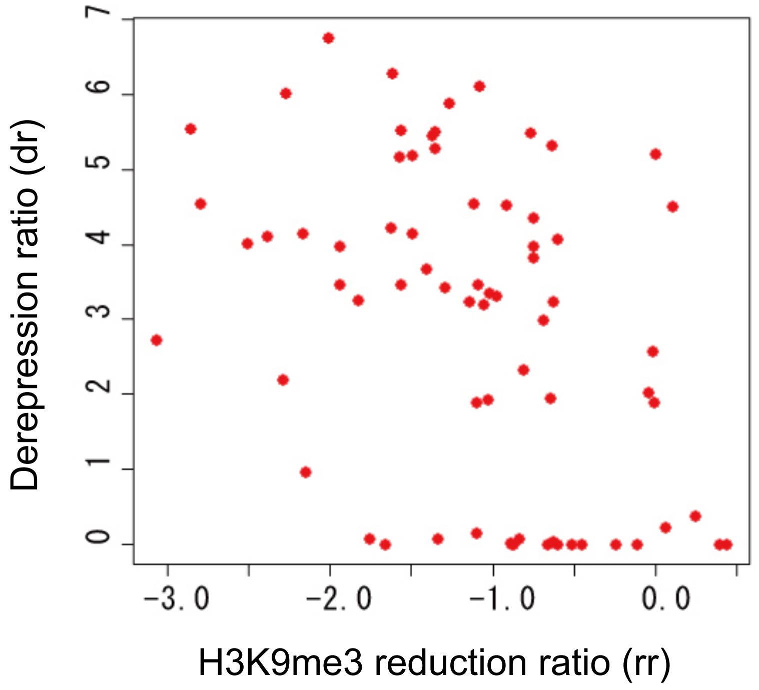

Supplementary Figure 4. Correlation between derepression ratio and H3K9me3 reduction ratio at VL30 (RLTR6_Mm) U3 I loci in *Setdb1* WT and KO iMEFs. The derepression ratio (dr) is calculated using the following equation with RNA-seq data. dr = log2((RPKM^{KO}+1)/(RPKM^{WT}+1)). H3K9me3 reduction ratio (rr) is calculated using the following equation with ChIP-seq data. rr = log2(RPKM^{KO}/RPKM^{WT}). Setdb1 data (red) shows two groups (dr and rr) are weakly correlated. Correlation coefficient is -0.42339 ($P < 6.16e-05$, Student's *t*-test). The rr mean of Setdb1 data is -1.3233.

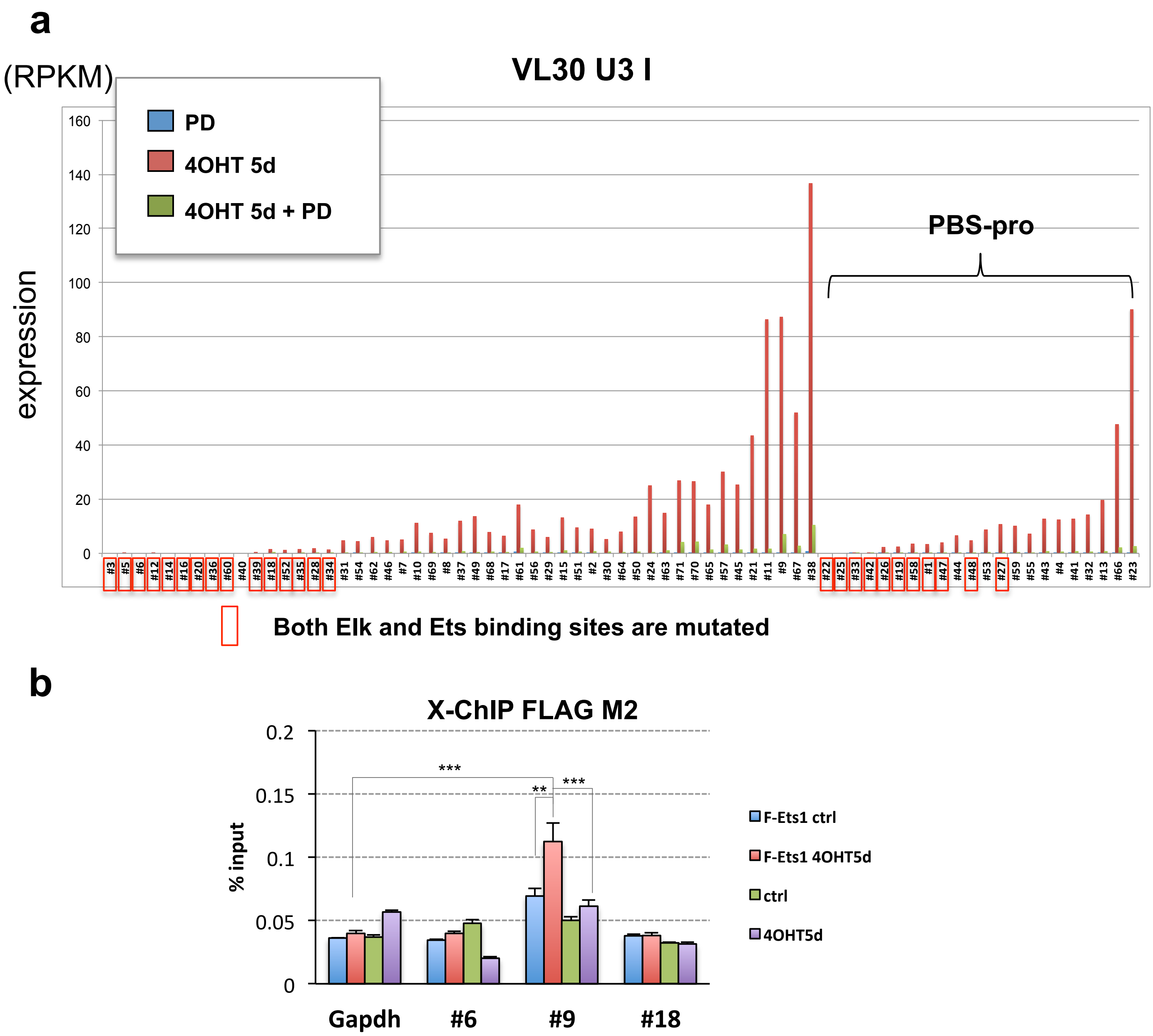

Supplementary Figure 5. Contribution of the MAPK pathway for VL30 U3 I derepression after Setdb1 depletion. (**a**) The derepression induced by loss of Setdb1 and the effect of a MEK inhibitor, PD325091 in each VL30 U3 I element (totally 71 elements) in *Setdb1* cKO iMEFs are shown. The number of mRNA-seq reads overlapping with a locus was divided by the length of the locus and normalized by million mapped reads (RPKM). 22 loci on the right side have typical PBS-pro near the 3‘ end of 5’ LTR. (**b**) Ets1 enrichment upon *Setdb1* KO at VL30 loci. ChIP-qPCR of FLAG tagged Ets1(F-Ets1) in the *Gapdh* promoter region and indicated VL30 elements of *Setdb1* cKO iMEFs stably expressing of FLAG-Ets1 or not. A representative of reproducible independent experiments (n = 3) is shown. Values are means ± s.d. from technical experiments (n = 3). **0.0005 < P < 0.005, ***P < 0.0005, Student's t-test.

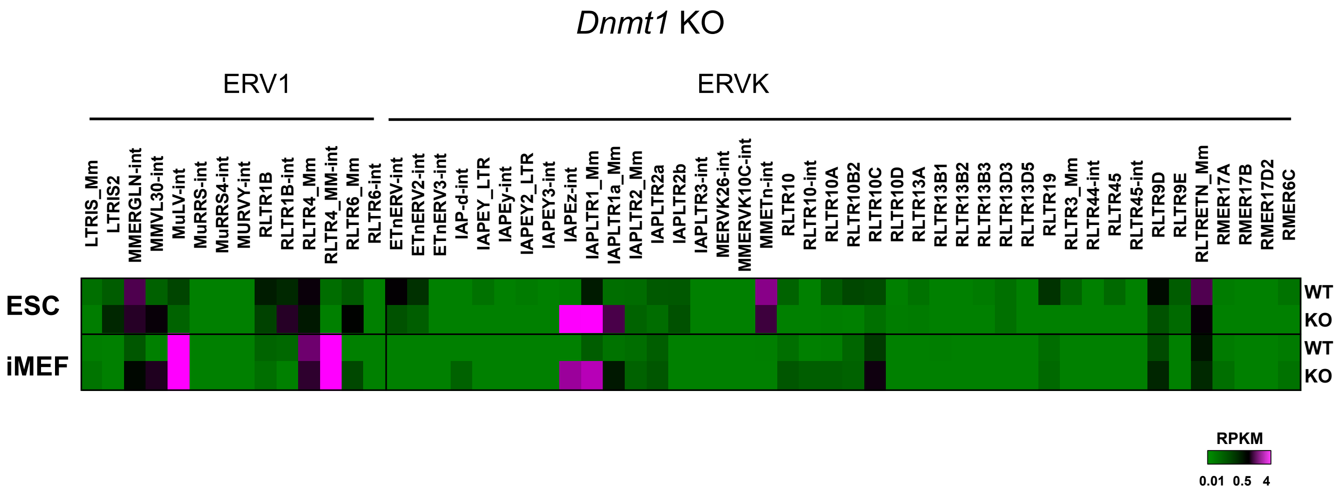

Supplementary Figure 6. Expression of ERV families in *Dnmt1* KO ESCs and iMEFs. RNA-seq data for *Dnmt1* cKO ESCs (without (WT) or day 6 after 4OHT treatment)[36] and *Dnmt1* KO or WT iMEFs (*p53-/-* background)[35] were reanalyzed. Heatmap indicates the magnitude of the RPKM value.

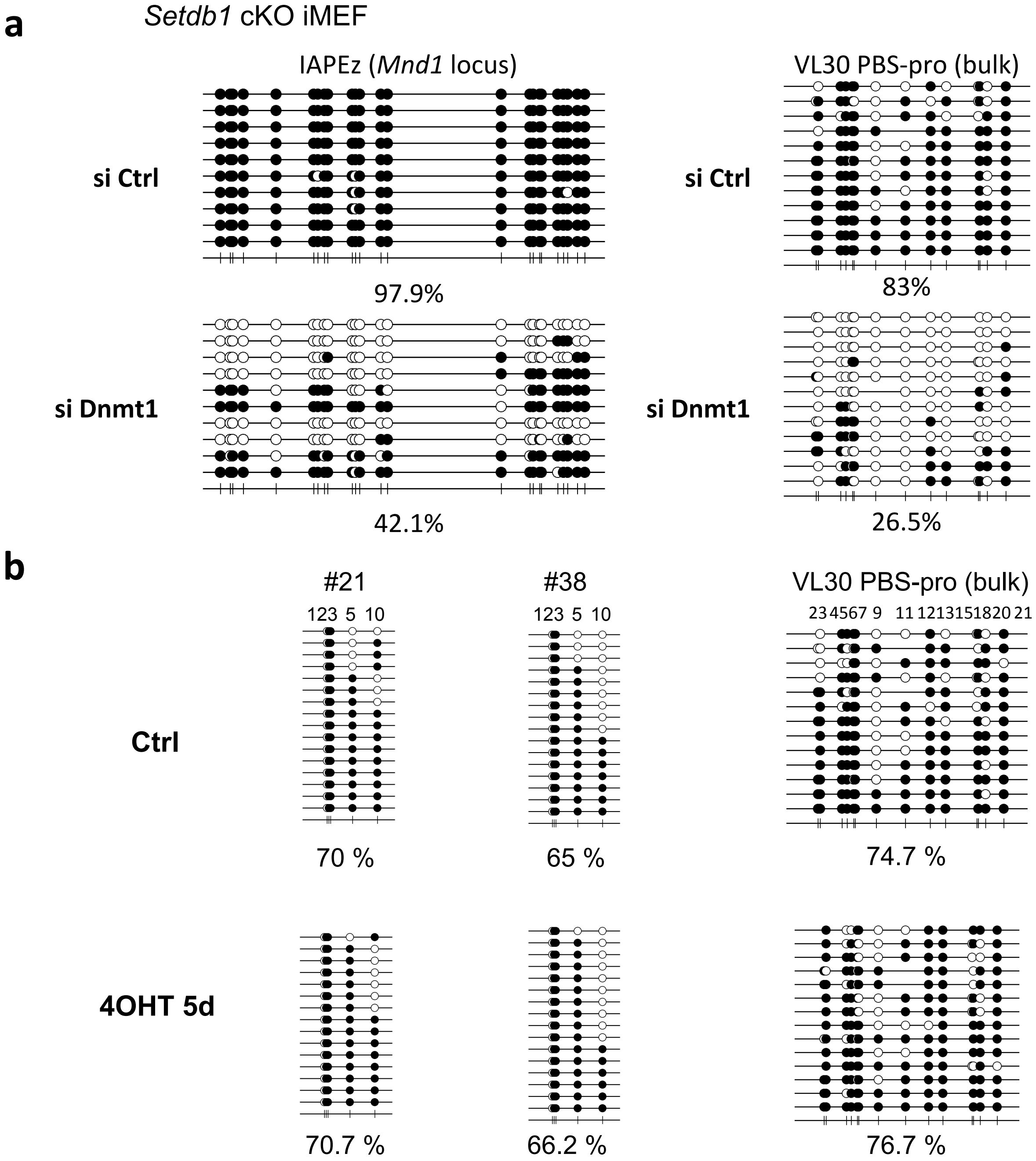

Supplementary Figure 7. DNA methylation analysis (**a**) Bisulfite sequencing analysis of the 5' LTR of IAPEz and VL30 in *Setdb1* cKO iMEFs transfected with control siRNA or siRNA against *Dnmt1* as described in Fig.4b. Open and filled circles indicate unmethylated and methylated (or hydroxymethylated) Cs in CG, respectively. (**b**) Bisulfite sequencing analysis of the 5' LTR of VL30 (individual elements of VL30 #21 and #38 (both non-PBS-pro) and bulk of VL30 PBS-pro elements) in *Setdb1* cKO iMEFs of control (Ctrl) and treated with 4OHT for 5 days (4OHT 5d). DNA methylation of 5' LTR (downstream of "TATAA" like box) was analyzed. The location of CG sites analyzed is shown in Supplementary Fig. 8.

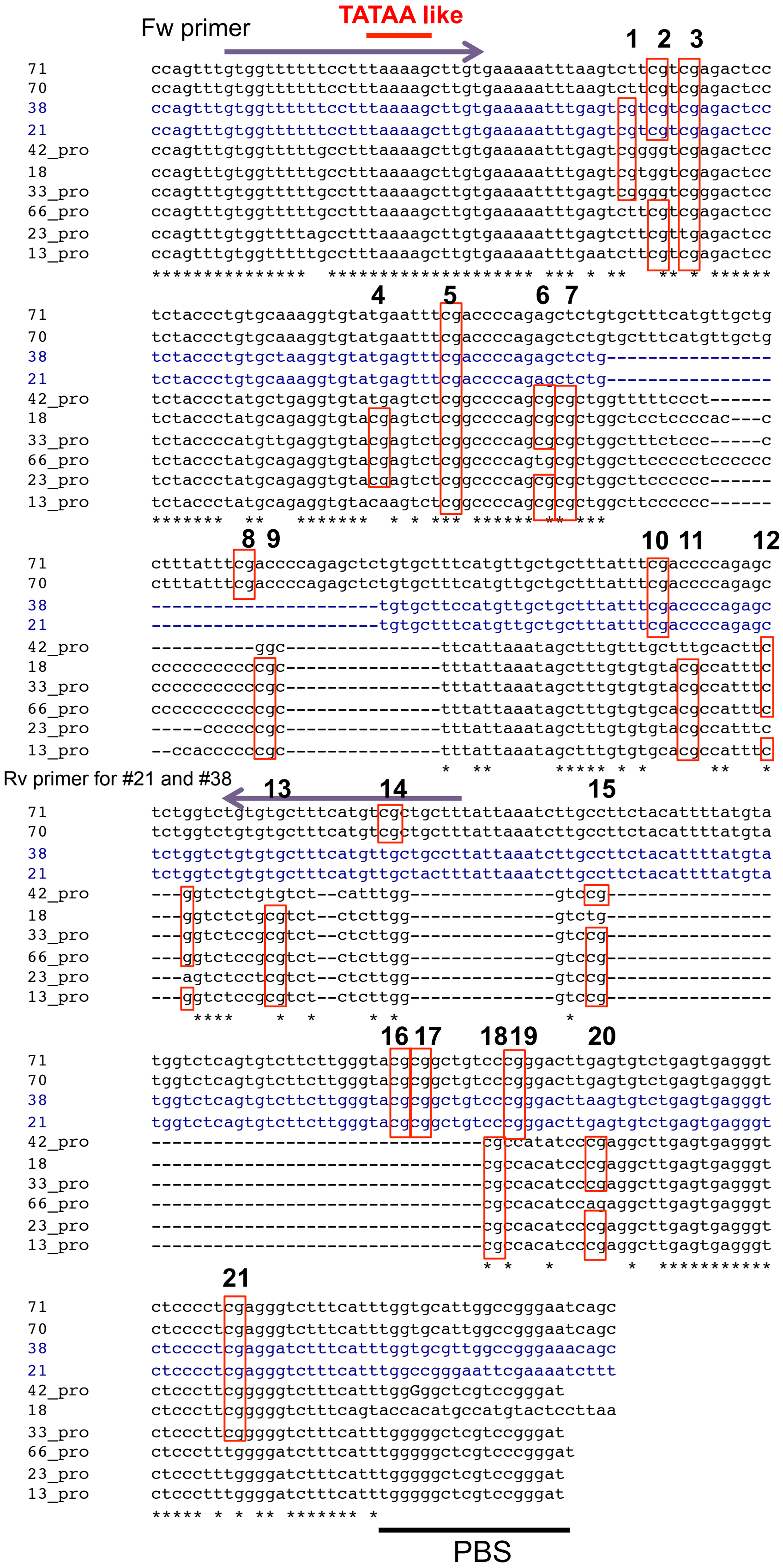

Supplementary Figure 8. Alignment of VL30 U3 class I sequences. Red boxes indicate CG sites

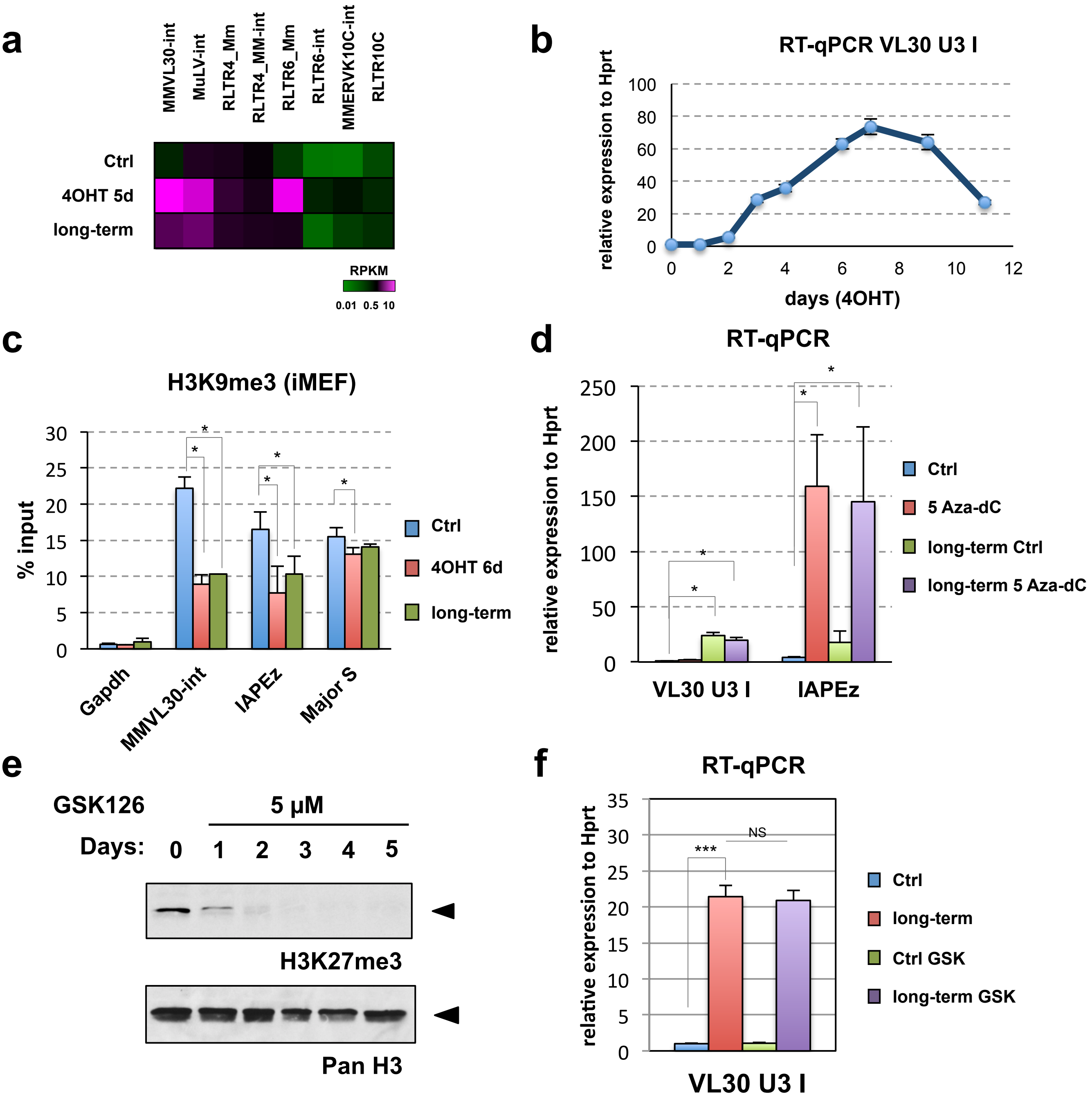

Supplementary Figure 9. Desensitization of VL30 expression in long-term cultured *Setdb1* KO iMEFs. (**a**) Expression of representative ERV families as measured by RNA-seq. Total reads were mapped to canonical proviral sequences. Intensity of colors indicates RPKM values of ERV families in iMEFs (untreated (Ctrl), 5-day treatment with 4OHT (4OHT 5d) and long-term culture (long-term)). (**b**) RT-qPCR analysis of VL30 U3 class I expression from day 0 to day 11 after 4OHT treatment. (n = 3 technical replicates). Error bars represent s.d.. (**c**) ChIP-qPCR of H3K9me3 at the indicated ERV and major satellite (Major S) loci of iMEFs (untreated, 6-day treatment with 4OHT and long-term culture). ($n \geq 2$ biological replicates). Error bars represent SD. *$P < 0.05$, Student's t-test. (**d**) RT-qPCR analysis of IAPEz and U3 class I VL30 with or without 2-day treatment with 5-Aza-dC (0.3 μM) in WT iMEFs and long-term cultured *Setdb1* KO iMEFs. Cells were harvested 4 days after the removal of 5-Aza-dC. (n = 2 biological replicates). Error bars represent s.d.. *$P < 0.05$, Student's t-test. (**e**) Efficiency of GSK126 for demethylation of H3K27me3 in iMEFs was examined by immunoblotting with anti-H3K27me3 antibody. (**f**) RT-qPCR analysis of VL30 U3 I expression in *Setdb1* cKO iMEFs treated with either 4OHT, GSK126 (GSK) alone or in combination with both (4OHT GSK) for 5 days. (n = 3 technical replicates). Error bars represent s.d.. ***$P < 0.0005$, Student's t-test.

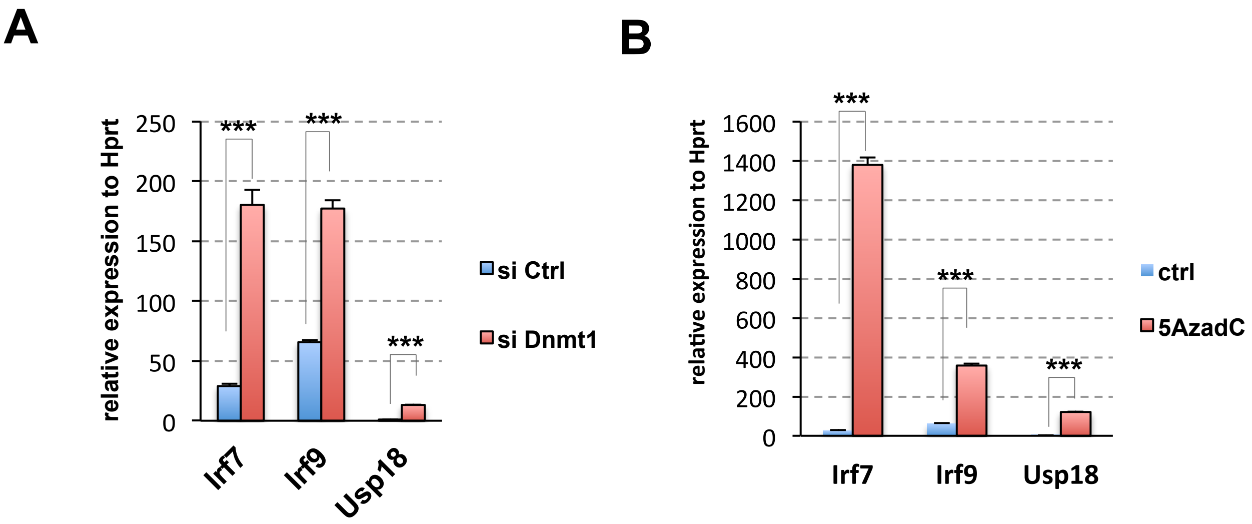

Supplementary Figure 10. Activation of IFN pathway-related genes by DNA demethylation. (a) RT-qPCR analysis of the IFN pathway related genes. *Setdb1* cKO iMEFs were transfected with siRNA against *Dnmt1* or control siRNA. **(b)** Treatment of 5-Aza-dC induces expression of the IFN pathway-related genes in iMEFs. iMEFs were treated with 0.2 μM of 5-Aza-dC for 2 days. RT-qPCR was performed at day 6. (n = 3 technical replicates). Error bars represent s.d.. ***$P < 0.0005$, Student's t-test.

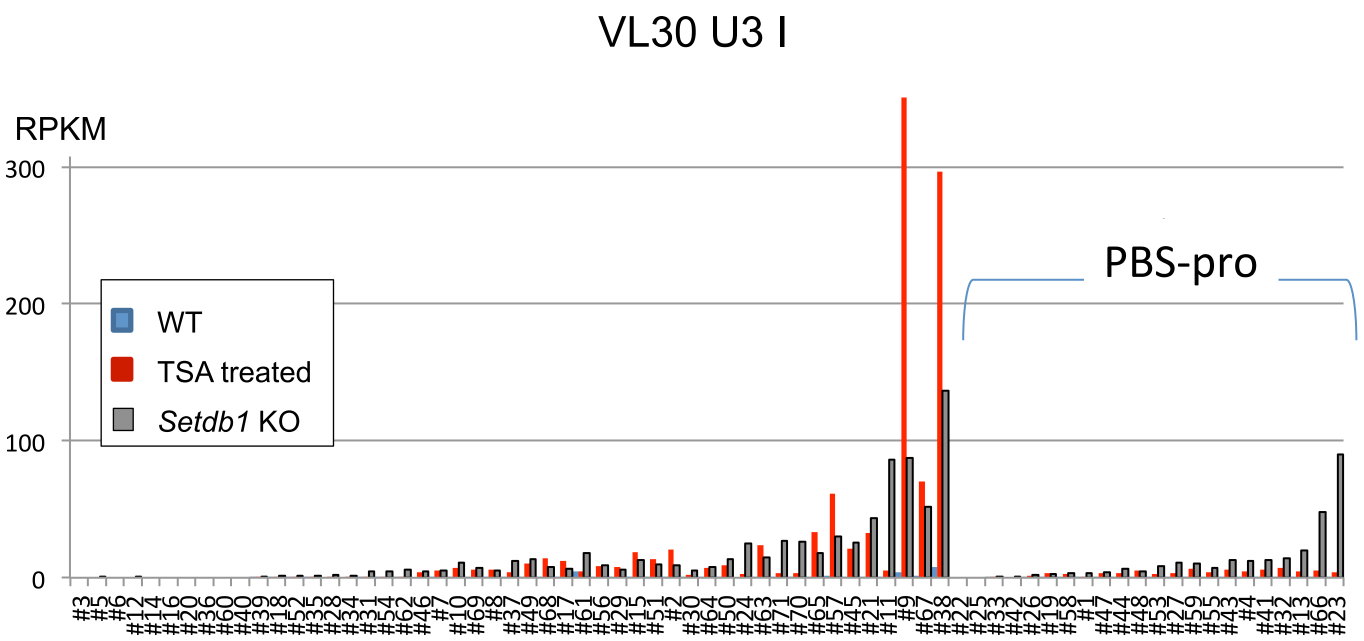

Supplementary Figure 11. HDAC inhibitor induces the expression of some elements of VL30. The derepression induced by TSA treatment or *Setdb1* cKO in each VL30 U3 I element (totally 71 elements) is shown. The number of mRNA-seq reads overlapping with a locus was divided by the length of the locus and normalized by million mapped reads (RPKM). 22 loci on the right side have typical PBS-pro near the 3' end of 5' LTR. The order of the elements on the horizontal axis is same as in Fig 3c. #9, #63 and #21 are same elements as NT_039207, NT_039649 and NT_039341 respectively described[52].

Fig. 5c

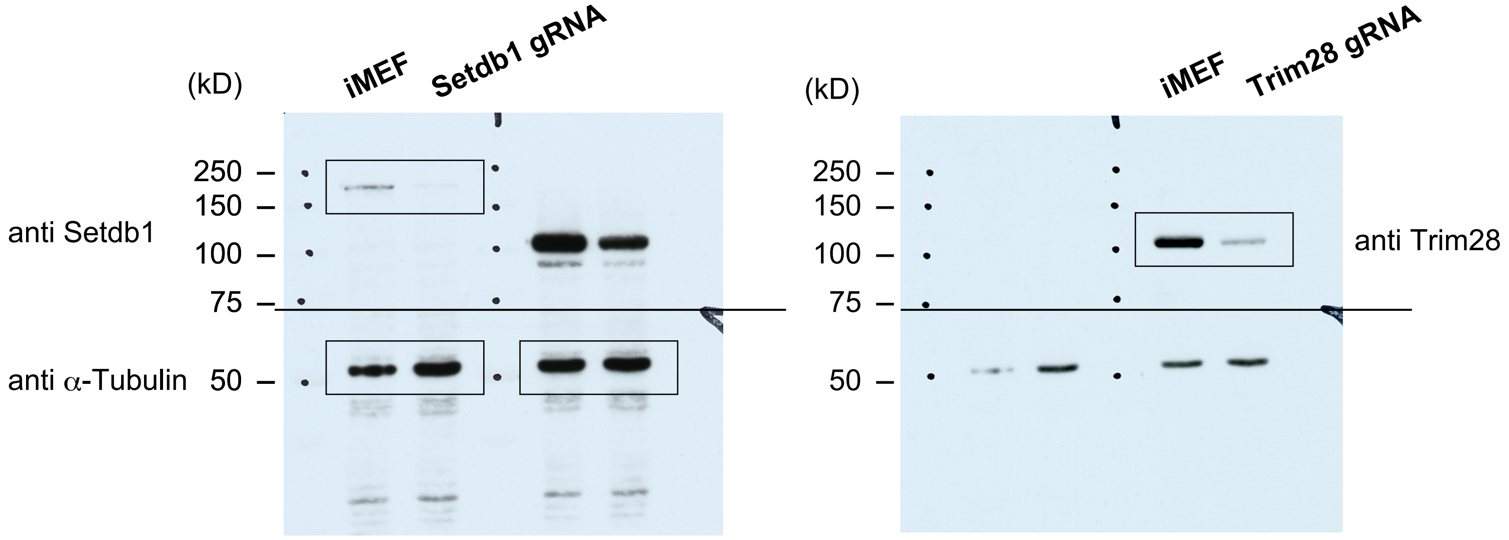

Fig. 5d

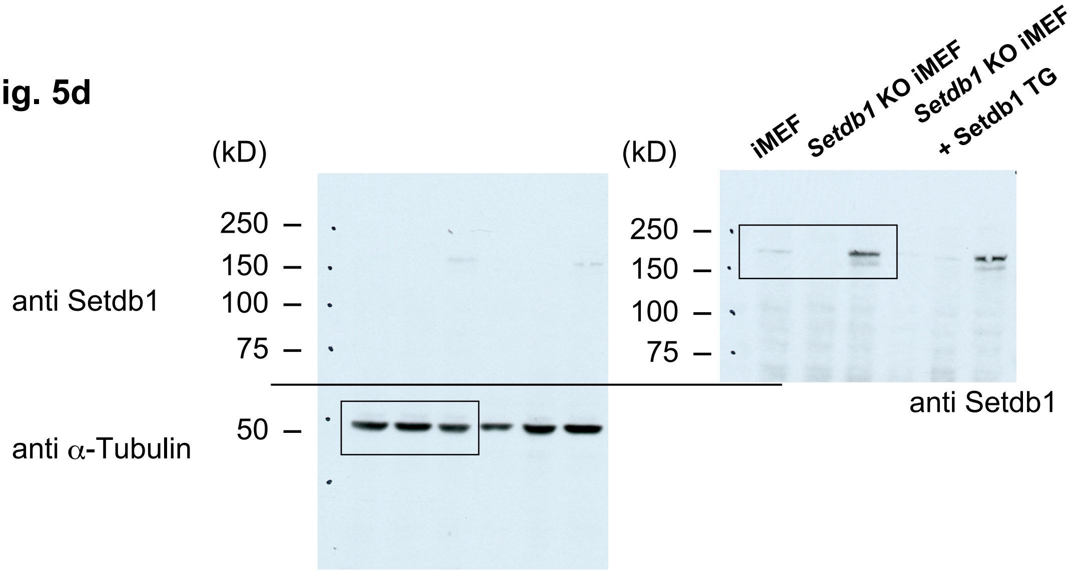

Supplementary Fig. 1b

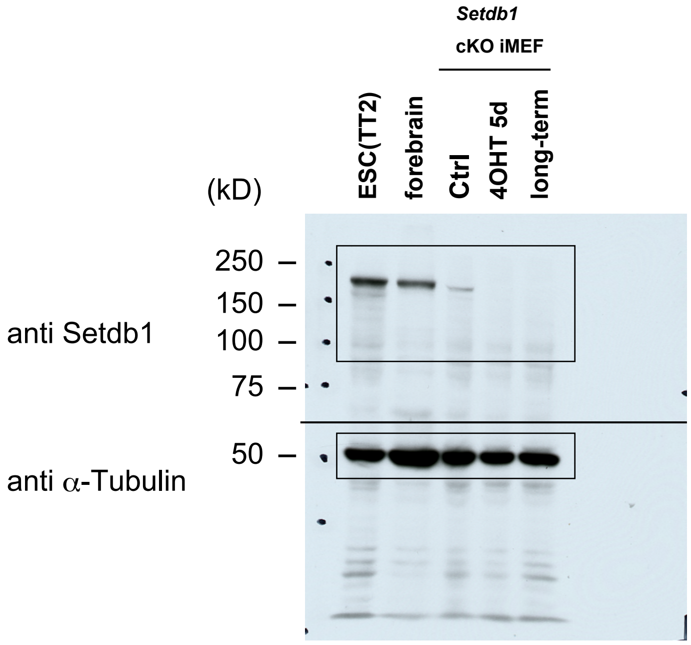

Supplementary Fig. 9e

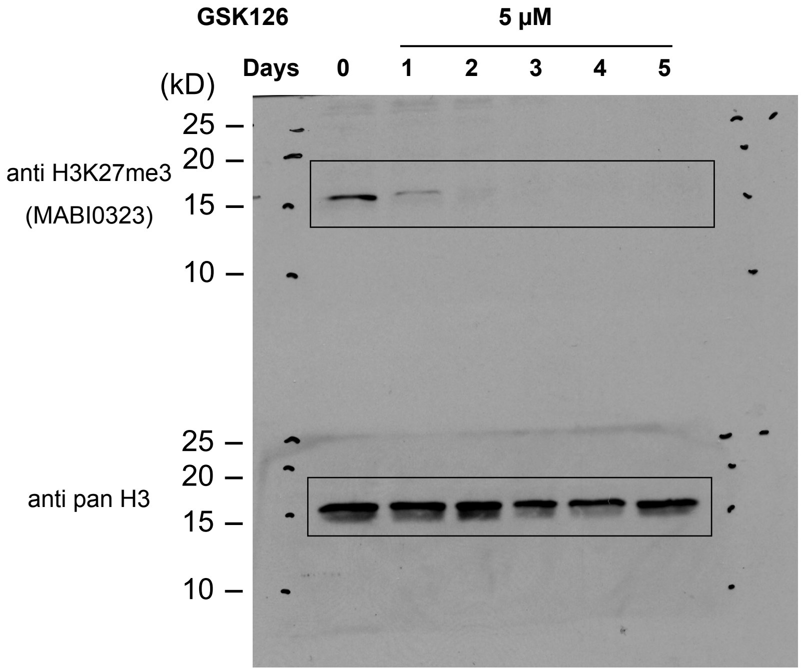

Supplementary Figure 12. Uncropped immunoblots for Fig. 5c, d, Supplementary Fig. 1b and Supplementary Fig. 9e

Supplementary Table 1

Primers used for RT-PCR and ChIP

Primer name	Application		Reference
Gapdh_ChIP F	ChIP-qPCR	ATCCTGTAGGCCAGGTGATG	3
Gapdh_ChIP R		AGGCTCAAGGGCTTTTAAGG	
MMVL30-int F	ChIP-qPCR	TCAACAGGCCAGATGTATTGC	
MMVL30-int R		ACAAACTGGGAGGGGGAAT	
IAPEz F	ChIP-qPCR/RT-qPCR	GCTCCTGAAGATGTAAGCAATAAAG	59
IAPEz R		CTTCCTTGCGCCAGTCCCGAG	
RLTR4.int.chr8 F	ChIP-qPCR/RT-qPCR	CATACTTCTGCCCCAGCTAA	60
RLTR4.int.chr8 R		CAGTAATCGGTGGTGAGGTC	
Major Satellite ChIP F	ChIP-qPCR	GACGACTTGAAAAATGACGAAATC	3
Major Satellite ChIP R		CATATTCCAGGTCCTTCAGTGTGC	
VL30_#6 F	ChIP-qPCR	TTGTTGCAAGGATGTCCAAA	
VL30_#6 F		GGGCTCCGAGGTGAAAAGTT	
VL30_#9 (NT_039207) F	ChIP-qPCR	AGGGCTCAGTGGTTATGGTCTTTT	52
VL30_#9 (NT_039207) R		AGGGGGAATGGGGAGGGAATAGG	
VL30_#18 F	ChIP-qPCR	TTGCTTCTTTATGCCACATGGT	
VL30_#18 F		GGGCTCCGAGGTGAAAAGTT	
Hprt_mmRT2 F	RT-qPCR	CAGGCCAGACTTTGTTGGAT	61
Hprt_mmRT2 R		TTGCGCTCATCTTAGGCTT	
VL30_U3I (LTR) F	RT-qPCR	AGATGTATTGCCAAACACAGG	28
VL30_U3I (LTR) R		AGGGGGAATGGGGAGGGAA	
VL30_U3II (LTR) F	RT-qPCR	GAACTCTTCCTCACCCCAGA	28
VL30_U3II (LTR) R		GAGGAGGAGTTCAGGAATGC	
Irf7_RT F	RT-qPCR	TGATCTTTCCCAGTCCTGCT	28
Irf7_RT R		TGCCTACCTCCCAGTACACC	
Irf9 F	RT-qPCR	TTCATCTATGGTGGCCGAGT	28
Irf9 R		ACGCCTCTGTCAAGCTGATT	
Usp18 F	RT-qPCR	CAGACGTGTTGCCTTAACTCC	62
Usp18 R		ACTCCGAGGCACTGTTATCC	
IAP_Mnd1 bis1st F	Bisulfite sequence	TTTTGGGTAAAAGTTTGTTTGTAAG	
IAP_Mnd1 bis2 F		GGGTATTTATGTAATAATTTGTG	
IAP_Mnd1 bis2 R		CCTTCTTAACAATCTACTTT	
VL30_pro BS F	Bisulfite sequence	GTGGTTTTTGTTTTTAAAAGTTTGTG	38
VL30_pro BS R		CCTTCTCTCCATCTCRAAAAAC	
VL30_21 BSP F	Bisulfite sequence (1st)	TGAAGAATGAAAAATTATTGGTTT	
VL30_21 BSP R		AAAACACACAAACCAAAACTCT	
VL30_38 BSP F	Bisulfite sequence (1st)	AAAAAATTATTGGTTTTTTGTGA	
VL30_38 BSP R		ATAAAAACACACAAACCAAAACT	
VL30_BS F	Bisulfite sequence (nested)	GTGGTTTTTTTTTTTAAAAGTTTGTG	
VL30_BS R		AAAACACACAAACCAAAACTCT	
gRNA sequences			
Setdb1	gRNA	GACTTACGTGGTCGGCAAAT	
Trim28	gRNA	GGTGCAGAGCCGCATGTATC	
