# Supplementary material for: A somatic role for the histone methyltransferase Setdb1 in endogenous retrovirus silencing
Source: Nat Commun. 2018 Apr 27;9:1683. doi: 10.1038/s41467-018-04132-9 (PMC5923290; doi:10.1038/s41467-018-04132-9)
Supplement: Supplementary file 3 — Description of Additional Supplementary Files [file 41467_2018_4132_MOESM3_ESM.pdf]

## Description of Additional Supplementary Files

**File Name: Supplementary Data 1**
**Description:** List of Up-regulated genes in Setdb1 KO iMEFs.

**File Name: Supplementary Data 2**
**Description:** H3K9me3 ChIP-seq reads on ERVs and LINEs in Setdb1 cKO iMEFs.

**File Name: Supplementary Data 3**
**Description:** Genomic distribution of each element of VL30 U3 class I. RPKM values of each element in Setdb1 cKO iMEFs (WT and KO (4OHT 5d) along with each sequences (1-300, red: mutated, blue: Elk site, light blue: AP-1 site and green: Ets site) were shown.

**File Name: Supplementary Data 4**
**Description:** Genomic positions of ERVs used for the analysis.
